# Song Competition Affects Monoamine Levels in Sensory and Motor Forebrain Regions of Male Lincoln's Sparrows (*Melospiza lincolnii)*

**DOI:** 10.1371/journal.pone.0059857

**Published:** 2013-03-26

**Authors:** Kendra B. Sewall, Samuel P. Caro, Keith W. Sockman

**Affiliations:** 1 Department of Biology, University of North Carolina, Chapel Hill, North Carolina, United States of America; 2 Curriculum in Neurobiology, University of North Carolina, Chapel Hill, North Carolina, United States of America; Utrecht University, The Netherlands

## Abstract

Male animals often change their behavior in response to the level of competition for mates. Male Lincoln's sparrows (*Melospiza lincolnii*) modulate their competitive singing over the period of a week as a function of the level of challenge associated with competitors' songs. Differences in song challenge and associated shifts in competitive state should be accompanied by neural changes, potentially in regions that regulate perception and song production. The monoamines mediate neural plasticity in response to environmental cues to achieve shifts in behavioral state. Therefore, using high pressure liquid chromatography with electrochemical detection, we compared levels of monoamines and their metabolites from male Lincoln's sparrows exposed to songs categorized as more or less challenging. We compared levels of norepinephrine and its principal metabolite in two perceptual regions of the auditory telencephalon, the caudomedial nidopallium and the caudomedial mesopallium (CMM), because this chemical is implicated in modulating auditory sensitivity to song. We also measured the levels of dopamine and its principal metabolite in two song control nuclei, area X and the robust nucleus of the arcopallium (RA), because dopamine is implicated in regulating song output. We measured the levels of serotonin and its principal metabolite in all four brain regions because this monoamine is implicated in perception and behavioral output and is found throughout the avian forebrain. After controlling for recent singing, we found that males exposed to more challenging song had higher levels of norepinephrine metabolite in the CMM and lower levels of serotonin in the RA. Collectively, these findings are consistent with norepinephrine in perceptual brain regions and serotonin in song control regions contributing to neuroplasticity that underlies socially-induced changes in behavioral state.

## Introduction

Animals must adjust their behavior according to changing and unpredictable environmental conditions, including variable social conditions. The monoamine neuromodulators play a pivotal role in mediating responses to changing conditions by modifying neural processes underlying behavioral plasticity [1]–[6]. Specifically, monoamines can modify neural selectivity and the efficiency of synaptic transmission to achieve shifts in behavioral state such as arousal, attention, motivation and mood [2], [4], [7], [8]. Though the monoamines have overlapping roles in regulating neuroplasticity, each monoamine is implicated principally in particular cognitive processes essential to adaptive changes in behavior. Norepinephrine is particularly involved in the regulation of attention and sensory processing central to memory consolidation and the optimization of behavior [4], [7]; dopamine is especially involved in motor control as well as reinforcement, reward anticipation and goal-directed behaviors [9]–[11]; and serotonin has been implicated in regulating diverse behaviors including memory formation and maintenance, sensory encoding, sensory-motor learning [12]–[14], sexual behavior and aggression [1], [3], [15].

**Figure 1:**
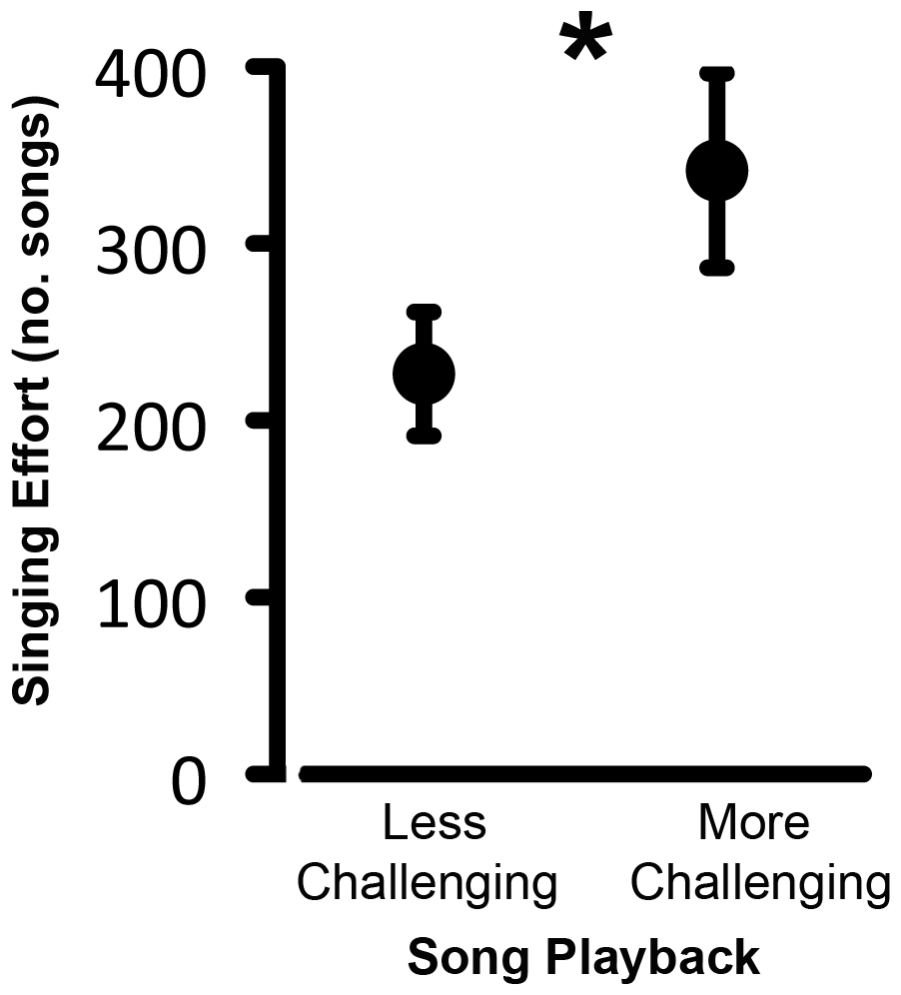
Effect of Prior Song Challenge on Singing Effort. The mean (± standard errors) number of songs males in the more challenging and less challenging treatment group produced the morning after the song playback ceased. The difference in song number reflects differences in males' competitive state because singing was not occurring in response to a playback stimulus. Figure modified from Sewall et al. (2010) with permission.

Understanding the coordinated roles of the monoamines in regulating adaptive shifts in social behavior requires presenting animals with a social context that elicits a change in behavioral state, but one which falls within the scope of naturally occurring behaviors. We previously demonstrated that simulating shifts in the competitiveness of the social environment, using playback of naturally variable songs, induced changes in the competitive behavioral state of territorial male Lincoln's sparrows (*Melospiza lincolnii*; [16]; Fig. 1). This research system provides an opportunity to examine the relationship between monoamine levels and socially-induced modulation of behavioral state. Here, using the same wild-caught male Lincoln's sparrows from the above-mentioned study, we examined the effect of natural variation in the competitiveness of the song environment on forebrain monoamine levels.

Male animals often must compete with one another for access to mates, and success in such male-male competition directly influences males' fitness. The level of challenge during mating and territorial contests changes, though, and in many songbirds variation in male-male competition is reflected by singing behavior [17], [18]. Several song features are reliably associated with measures of male condition [19]–[25], permitting prospective mates and competitors to evaluate individuals based on their songs [18], [26]–[28]. For example, songs that are longer or more complex can be associated with higher-quality and thus more challenging competitors [19], [22], [23], [25]. When territorial male songbirds are presented with songs associated with greater challenge in brief playback experiments, they respond more aggressively, which includes increasing the number of songs they produce [29]–[32].

**Figure 2:**
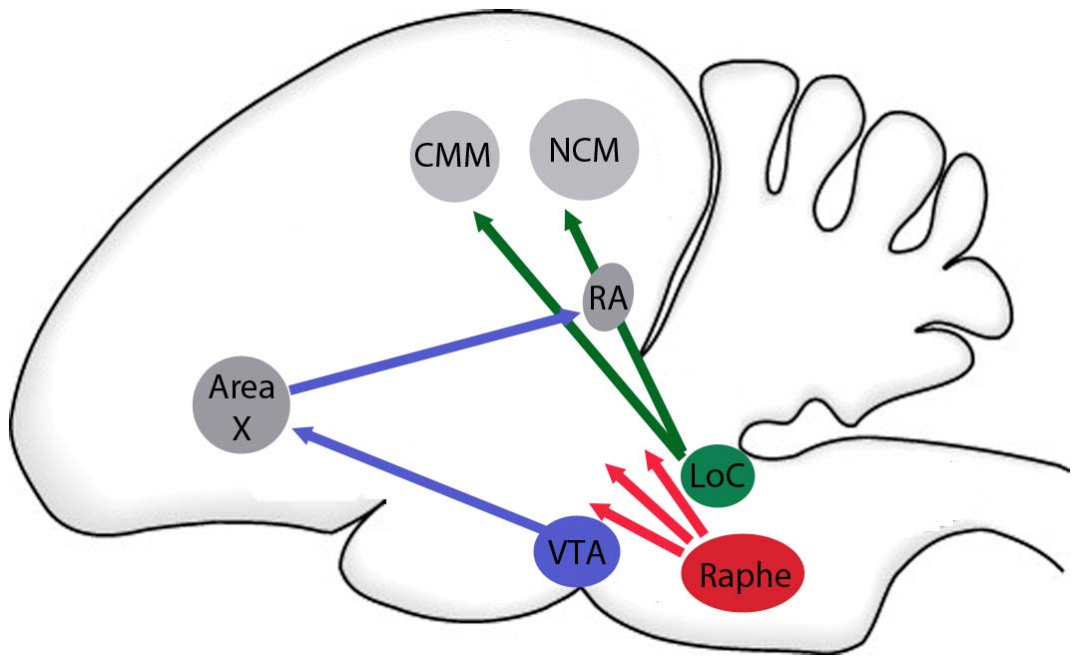
Auditory, Song Control and Monoamine Centers of the Avian Brain. A diagram of the auditory processing, song production and monoamine centers of the brain examined in this study. Though dopaminergic, noradrenergic and serotonergic cells are found in all of the brain regions of interest, the figure illustrates our approach of focusing on a subset of monoaminergic correlates of behavior. Green: noradrenergic projections, blue: dopaminergic projections, red: serotonergic projections.

Similarly, male Lincoln's sparrows exposed to persistent playback of more challenging songs (songs that are longer and more complex than average for the population, see methods) for a week increase the number of songs they produce (i.e., their competitive effort) more than males exposed to less challenging (shorter and less complex than average) songs ([16]; Fig. 1). The effects of exposure to songs of varying level of challenge persist after playbacks have ended, indicating that these behavioral differences reflect changes in the males' competitive states [16], [33], [34]. Given that socially-elicited changes in behavioral state can be mediated by monoamine-dependent neural plasticity, we examined the relationship between social challenge and the levels of particular monoamines in two forebrain networks implicated in song perception and the modulation of song motor output (Fig. 2). Because norepinephrine is hypothesized to modify the sensitivity of neurons in the avian auditory forebrain [5], [35]–[39], and because the auditory forebrain receives strong noradrenergic innervation [40], we quantified the levels of norepinephrine and it's primary metabolite in two areas that mediate the perception of conspecific songs, the caudal medial nidopallium (NCM) and the caudal medial mesopallium (CMM; [41]–[46]; Fig. 2). Similarly, because dopamine is implicated in regulating context-specific singing through action in nodes of the song control system [11], [47], [48], and because these brain regions receive particularly strong innervation from a dopaminergic center (the ventral tegmental area; [49]–[54]; Fig. 2), we measured the levels of dopamine and its primary metabolite in two nuclei of the song control pathway specifically implicated in context-dependent singing, area X and the robust nucleus of the arcopallium (RA; [55], [56]). We measured the levels of serotonin and its primary metabolite in all four brain regions of interest, because much of the avian forebrain receives strong serotonergic innervation from the raphe nuclei ([57]; Fig. 2) and serotonin is implicated in the regulation of perception [3], [58]–[61], as well as in regulating sensory-motor behaviors including vocalizing [62]–[64], and aggression [1], [15], [65], which could include singing. Finally, we examined the relationship between the monoamines, the song playback treatment, and recent song output to determine if monoaminergic activity was explained by recent motor output, in addition to the level of song challenge. The primary goal of this study is to identify the monoamine changes across integrated brain regions, which may underlie socially-induced shifts in behavior. This work could lay the groundwork for future comparisons of monoamine expression in wild populations. Additionally, the approach of describing concerted monoaminergic changes across brain regions emphasizes the importance of examining integrated changes throughout the brain and generates hypotheses about monoaminergic function under naturalistic conditions, which may serve as the basis for future manipulations of these brain substrates.

## Materials and Methods

### Ethics statement

The U.S. Department of the Interior's Fish and Wildlife Service (permit MB099926), the U.S. Department of Agriculture's Forest Service (authorization COL258), the State of Colorado's Department of Natural Resources Division of Wildlife (license 06TR1056A2), the Town of Silverton, Colorado, USA, and the University of North Carolina at Chapel Hill Institutional Animal Care and Use Committee (protocol 05-138.0-A) each granted permission to conduct the procedures described in this study.

### Experimental procedures and subjects

We presented adult male Lincoln's sparrows with unique sets of either more challenging or less challenging songs (see song playback treatment), played back repeatedly for 7 consecutive days, to elicit a change in competitive behavior. On the 8^th^ morning, after playback ceased, we collected the males' brains and used HPLC to measure levels of key monoamines and their primary metabolites from tissue samples from auditory processing and song control brain regions. Specifically, on 12 May 2008, close to the start of the breeding season for this species, we initiated the study by moving 18 male Lincoln's sparrows between the ages of 1–2 years from outdoor aviaries at the University of North Carolina at Chapel Hill to indoor cages. We had captured these males in the wild at approximately 8 days of age, hand-fed them, and tutored them as a single group using recorded song and live adult males. For the entire study we provided the birds with *ad libitum* food (Daily Maintenance; Roudybush, Woodland, CA, USA) and water. Once in individual cages, we held the subjects on a 16 hour light and 8 hour dark photoperiod (lights on at 05:00 and off at 21:00 EDT) for 2 weeks to maintain their reproductive-like physiological state [66]. Because we had only eight experimental set-ups, we designed the study as two balanced replicates, which occurred over two consecutive weeks. At 09:00 on 26 May 2008, we randomly assigned and transferred each of the first 8 subjects to eight individual cages within each of eight sound attenuation chambers (58×41×36 cm, Industrial Acoustics Company, New York, NY, USA). Each chamber had a fan-driven ventilation system and a light that we used to maintain the above light-dark schedule. We equipped each chamber with (1) an omni-directional microphone (Senheiser ME 62, Old Lyme, CT, USA) plugged into an eight-line recording interface (PreSonus FP10, Baton Rouge, LA, USA) and a computer running Sound Analysis Pro II software (SAP Version 2.062; [67]) and (2) a speaker (Pioneer TS-G1041R, Tokyo, Japan) plugged into an individual amplifier (Audiosource Amp 5.1A, Portland, OR, USA) attached to an eight-channel interface (M-Audio Delta1010, Irwindale, CA, USA) and a computer running Pro Tools M-Powered playback software (version 7.1, M-Audio, Irwindale, CA, USA). We permitted the males to acclimate to the chambers until 06:00 the next day, when we began to play each male songs from one of two treatments – either songs that were less challenging or more challenging (see song playback treatments, below). We assigned males to chambers such that subjects of each treatment were spatially interspersed throughout the room. We exposed the males to these song treatments and collected audio recordings from these subject males for 7 days. On the eighth day we provided no playback but continued collecting audio recordings of the subjects until 09:00, when we began rapidly decapitating and removing the brain of each male. All brain removal was complete by 10:30.

Using previously described protocols [33], we fixed one hemisphere (alternating left and right between subjects within each treatment group) in 5% acrolein, saturated it with 30% sucrose for cryoprotection, froze it on dry ice and held it at −80°C for approximately two weeks until Nissl staining was conducted. The second hemisphere was fresh frozen on dry ice and held at −80°C until brain regions were micropunched and HPLC was conducted (ca. 18 wks, see below). We repeated these procedures with the second session of 8 males, beginning on 4 June 2008. During this second session, one male from each treatment group was found dead on the second day of playbacks. Two new males were added to the study beginning 6 June 2008, resulting in a third session that consisted of only two subjects, one from each treatment group, and ended 2 days after the second session.

### Song playback treatments

For the song playbacks, we used two sets of 48 recordings each (96 songs in total) from a library of songs collected from the subjects' natal meadow. We initially categorized each of the recorded songs used in this study as being either higher-quality (longer in duration and more complex based on their containing more syllables and more phrases), or lower-quality (shorter in duration and less complex, containing fewer syllables and fewer phrases), than average for the population [16]. This categorization is biologically relevant as, in an earlier experiment, female Lincoln's sparrows showed greater behavioral activity in response to playback of the set of songs we had categorized as higher-quality, compared to the set of lower-quality songs [68]. Given that males use song to attract and compete for females, songs preferred by females are presumably more challenging to male competitors. In the present experiment we refer to the set of higher-quality songs that females were more responsive to as more challenging songs and the set of lower-quality songs as less challenging songs to emphasize that the signaler and receiver are both males and that song playback reflects a social challenge. We chose to expose males to these two song playback treatments because we are interested in how natural variation in song challenge is transduced into behavioral and brain responses. We did not include a “no-song” or "heterospecific-only-song" treatment group because isolation from conspecific song is not a natural condition for Lincoln's sparrows in this reproductive state and would be expected to elicit abnormal behavioral and brain responses that would be inappropriate to assume as base-line values.

We exposed each male subject to either six unique songs from the more challenging stimulus set or six unique songs from the less challenging stimulus set. We used six songs per male to mimic different competitive environments, as may occur on a breeding meadow for this species, rather than challenge from a single competitor. The songs we played each subject were produced by at least two free-living males, neither of which provided recordings for the tutoring phase mentioned above. To maximize the generalizability of our study [69], [70] we used the playback recordings from each free-living male for no more than one subject in each of the two treatment groups. In some cases a wild male's higher-quality songs were played to a subject in the more challenging treatment and his lower-quality songs were played to a subject in the less challenging treatment. It was essential to present each subject with a unique set of recorded songs because the number of stimulus sets is the effective sample size [70]. We played songs back at 70 dB 5 cm from the speaker, following a pattern of intense morning singing and intermittent afternoon/evening song (9 hr per day at an average rate of approximately 40 songs per hr). To ensure that the total duration of song each day was identical between treatment groups, and thus that we could conclude that any behavioral differences were elicited by the level of challenge and not the amount of song males heard, we included additional repetitions of less challenging songs, which tend to be shorter (see above), as necessary. Therefore, the treatments differed not only in their song quality, but also in their song repetition rates, with the more challenging playback treatment having slightly lower song repetition rates.

As part of the aforementioned study [16] we quantified the subjects' singing behavior by counting the number of songs each male produced from 05:00–09:00 each day, including the morning after playback stopped. We found that all males increased their singing effort throughout the week, but that males exposed to more challenging songs increased singing effort more quickly and to a much greater degree, resulting in an almost three-fold difference between groups in their singing rates on the last day of playback [16]. Further, males exposed to more challenging songs had approximately a 50% higher singing rate on the morning after playback ceased than males exposed to less challenging songs ([16]; Fig. 1). It is important to note that differences in singing behavior on the day after playback stopped are not reflective of real-time responses to playback stimuli. Rather, this behavioral difference reflects changes in behavioral state resulting from the prior week of experience with competitors' songs. However, at the time of brain collection the subjects' in the two treatment groups differed in both their competitive state and their very recent singing behavior. Therefore, we examined the simultaneous contributions of both the playback treatment (which elicited the change in singing over the entire week) and measures of each individual's most recent singing behavior to variation in monoamine measures. This approach permitted us to determine if brain differences reflected differences in the song treatment regardless of recent behavior (see Statistical procedures).

### Tissue preparation and quantification of monoamines, metabolites and protein

**Figure 3:**
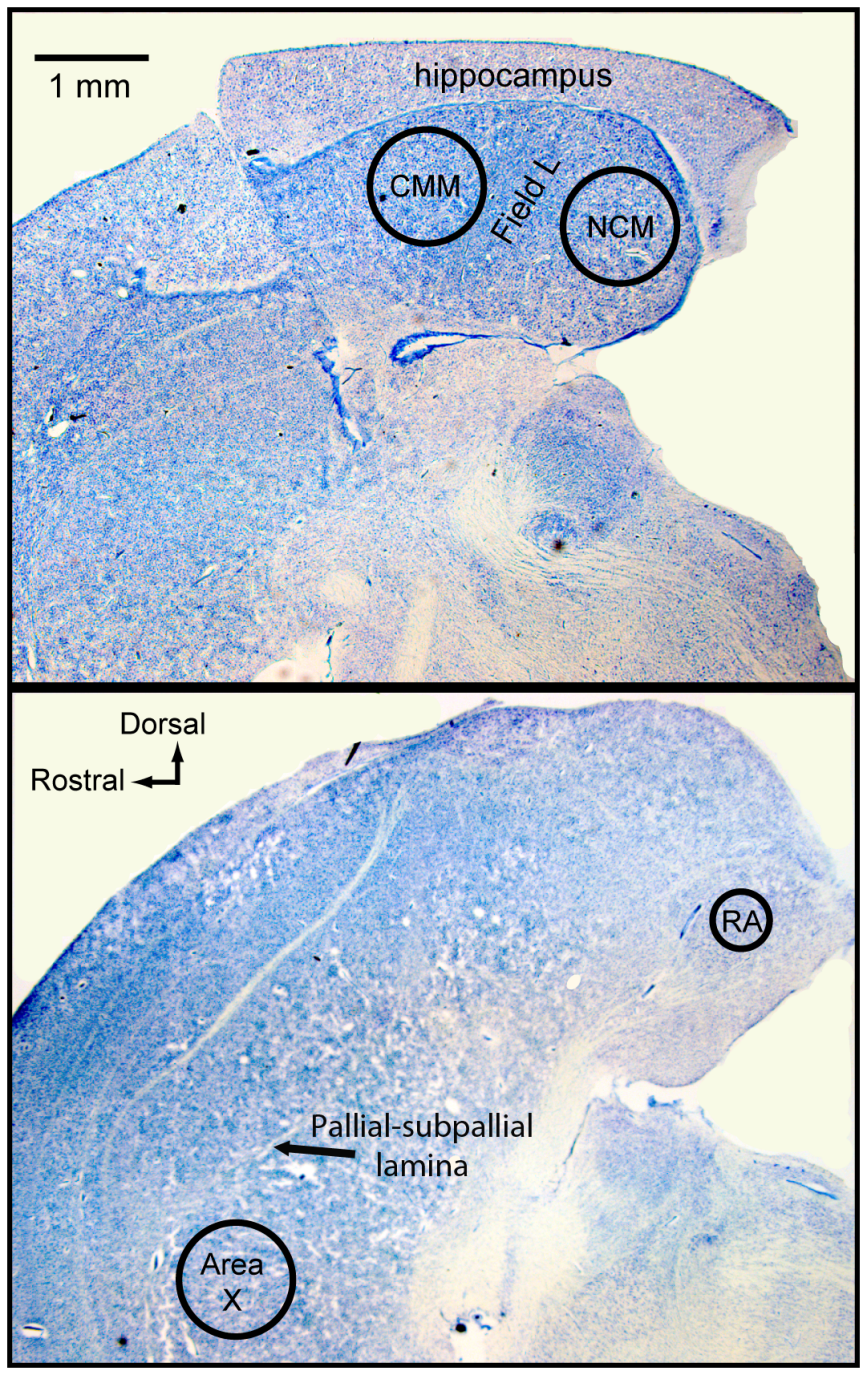
Placement of Tissue Punches. Photomicrographs of sagittal brain sections approximately 300 µm (upper panel) and 900 µm (lower panel) from the midline illustrating where micropunches of tissue were taken to quantify levels of norepinephrine, dopamine, serotonin, and their primary metabolites in the caudomedial mesopallium (CMM), the caudomedial nidopallium (NCM), area X, and the robust nucleus of the arcopallium (RA; lower image). Images generated for illustration only.

We sectioned the frozen, non-fixed hemisphere from each subject at 300 µm in the sagittal plane in a cryostat. We thaw mounted sections onto glass microscope slides and rapidly refroze the tissue on dry ice. Using micropunches (Fine Science Tools, Foster City, CA, USA), we took one tissue sample from each of four brain regions – the NCM and the CMM of the auditory telencephalon; the principal nucleus of the anterior forebrain pathway of the song control system, area X, and the principal nucleus of the motor pathway, RA. We chose brain sections containing each region based on boundaries defined by Nissl-staining ([33] for protocol) in sections from the alternate, fixed hemisphere and comparison with a zebra finch atlas [71]. Although inter-hemispheric differences in anatomy and plane of section could lead to errors when using one hemisphere (the Nissl stained one) to guide dissection in the other, we used punches with diameters well below the diameters of the brain regions of interest to ensure that we included only tissue that was within the targeted brain region. Further, we selected areas to sample that were bounded by visible neuroanatomical markers in fresh frozen tissue (i.e., RA, area X) or that were sufficiently large that we were confident that a tissue punch would be well within the bounds of the region (i.e., the CMM, the NCM) as defined by the Nissl-stained contralateral sections. We collected 1-mm-diameter punches from the center of both the NCM and the CMM, the boundaries of which have been described [36], [72], in the most medial brain section (Fig. 3). We sampled area X by taking one 1-mm punch from each of two consecutive sections that were 300–900 µm lateral to the midline, and RA by taking one 0.7-mm punch from each of two consecutive sections 1500–2100 µm lateral to the midline (Fig. 3). We expelled tissue punches into 1.9 mL polypropylene microcentrifuge tubes, froze them on dry ice and stored them at −80°C until assay (ca. 6 weeks). Immediately before assay, we added 125 µL of mobile phase containing 1 pg/µL of isoproterenol to each tube containing a tissue micropunch. We sonicated the samples and then centrifuged them at 16,000 *g* for 16 min at 4°C. We drew off the supernatant and transferred it to an autosampler tube; 10 µL of supernatant from each sample was injected into the HPLC system.

In addition to quantifying the amount of norepinephrine in the auditory forebrain regions, dopamine in the song control nuclei, and serotonin from all of the brain regions of interest, we also quantified the amount of the monoamine principal metabolites, 3-methoxy-4-hydroxy-phenylglycol (hereafter norepinephrine metabolite), 3,4-dihydrophenylacetic acid (hereafter dopamine metabolite), and 5-hydroxyindolacetic acid (hereafter serotonin metabolite). We used an HTEC-500 complete stand-alone HPLC-ECD system (Eicom, San Diego, CA, USA) coupled with a Midas autosampler (Spark Holland, Netherlands). We separated compounds using an Eicompak SC-3ODS column (Eicom) and used a mobile phase (pH 3.5) consisting of citric acid (8.84 g), sodium acetate (3.10 g), sodium octyl sulfonate (215 mg), EDTA (5 mg), methanol (200 mL) and ultra pure water (800 mL; all compounds, Sigma-Aldrich, St. Louis, MO, USA). We maintained the electrode potential at 750 mV with respect to the Ag/AgCl reference electrode. We prepared two standards with 1 pg/µL and 10 pg/µL of each of the 6 compounds of interest and used these two standard solutions to run a two-point standard curve at the beginning of each sample run (compounds listed above). We also included an internal standard, isoproterenol (Sigma-Aldrich), in each standard solution and tissue sample to identify any preparations from which sample was lost; no samples had significantly lower amounts of internal standard than expected.

Monoamines can be rapidly broken down into metabolites once they are secreted into the synapse and therefore their primary metabolites may serve as indices of monoamine metabolism. However, monoaminergic activity is a function of availability within the synapse, which is regulated by the rate of monoamine secretion and re-uptake, as well as catabolism. Thus, quantities of the monoamines themselves may reflect the amount of neuromodulators synthesized and stored pre-synaptically, or bound by metabolic or re-uptake enzymes within the synaptic cleft but not yet broken down or reabsorbed [73]. We quantified both the amounts of monoamines and their metabolites using high-pressure liquid chromatography with electrochemical detection, in an effort to understand how both monoamine availability and breakdown (hereafter referred to generally as monoaminergic activity) differed between the treatments. It should be remembered that monoaminergic activity results from the coordination of multiple cellular mechanisms, the subtlety of which cannot be captured by this experimental approach.

We calculated the amounts of each monoamine and metabolite by comparing the areas of the peaks of the compounds within each sample to those obtained from the two standard solutions that we used to generate the standard curve, using the peak area ratio function in PowerChrom software (eDAQ, Colorado Springs, CO, USA). Some peaks were not measurable and were omitted from the analysis (see degrees of freedom in Table 1). We then measured the protein content of each sample by dissolving the remaining protein pellet in 0.2 *M* NaOH (25 µL for 0.7 mm punch samples, 50 µL for 1 mm punch samples) and performing a Bradford protein-dye binding assay (Quickstart Bradford Protein Assay, Bio-Rad, Hercules, CA, USA) with bovine serum albumin as a standard on a µQuant microplate spectrophotometer (BioTek, Winooski, VT, USA). In a few cases the accuracy of the protein assay was poor. Because we did not have enough sample to repeat the assay, the amount of protein was estimated as the average amount of protein in the other samples from that brain region. This is an acceptable estimation because a standard micropunch was used and there was relatively little variation in protein quantities across tissue samples from a given brain region (e.g., 17 µg±4 µg in 0.7 mm punches from RA). We report the amounts of each compound of interest per mg of protein in the sample.

**Table 1:** Song playback and monoamine levels.

	Estimate	SEM	DF	t	*P*	Estimate	SEM	DF		t	*P*
NCM											
Norepinephrine						Norepinephrine metabolite					
Intercept	511.956	83.785	7	6.110		192.192	30.017		7	6.403	
Level of song challenge	11.162	46.522	6	0.240	0.818	53.467	64.461		5	0.829	0.445
Recent singing	−29.126	12.273	6	−2.373	0.055*	−9.566	6.679		5	−1.432	0.211
Serotonin						Serotonin metabolite					
Intercept	1218.784	205.999	7	5.916		390.097	81.743		7	4.772	
Level of song challenge	295.378	226.932	5	1.302	0.250	77.006	83.812		6	0.919	0.394
Recent singing	−65.574	41.979	5	−1.562	0.179	−38.028	15.948		6	−2.384	0.054*
CMM											
Norepinephrine						Norepinephrine metabolite					
Intercept	352.316	54.772	7	6.432		157.515	12.541		7	12.560	
Level of song challenge	−1.997	53.669	6	−0.037	0.971	42.639	9.963		5	4.280	0.008
Recent singing	−7.307	−7.307	6	−0.627	0.558	−4.837	2.448		5	−1.976	0.105
Serotonin						Serotonin metabolite					
Intercept	1560.179	342.115	7	4.560		458.762	75.893		7	6.045	0.001
Level of song challenge	352.239	399.113	6	0.883	0.411	70.671	74.064		6	0.954	0.377
Recent singing	−80.477	68.015	6	−1.183	0.282	−39.538	10.069		6	−2.192	0.070*
Area X											
Dopamine						Dopamine metabolite					
Intercept	2501.745	634.318	7	3.945		3704.655	622.71		7	5.949	
Level of song challenge	12.276	759.228	6	0.017	0.988	−382.886	1149.7		5	−0.333	0.753
Recent singing	−0.861	1.361	6	−0.633	0.550	3.572	1.862		5	1.919	0.113
Serotonin						Serotonin metabolite					
Intercept	70.670	12.674	7	5.576		66.553	22.639		7	2.940	
Level of song challenge	5.155	18.726	6	0.276	0.792	−11.264	18.087		5	−0.623	0.561
Recent singing	−0.026	0.030	6	−0.858	0.424	0.002	0.021		5	0.089	0.933
RA											
Dopamine						Dopamine metabolite					
Intercept	478.072	106.086	7	4.506		131.799	22.375		7	5.891	
Level of song challenge	−231.336	97.502	6	−2.373	0.055*	−16.104	16.946		6	−0.950	0.379
Recent singing	0.020	0.190	6	0.104	0.920	−0.063	0.038		6	−1.654	0.149
Serotonin						Serotonin metabolite					
Intercept	859.220	176.674	7	4.863		174.128	35.065		7	4.966	
Level of song challenge	−435.568	163.936	6	−2.657	0.038	−33.008	33.857		6	−0.975	0.367
Recent singing	0.065	0.217	6	0.297	0.776	−0.093	0.050		6	−1.852	0.113

Effects of the song playback and a subject's own recent singing behavior on amounts of three monoamines and their primary metabolites (measured as pg/mg protein) in auditory processing and song control regions of the forebrain of male Lincoln's sparrows. Level of song challenge (i.e., treatment) was coded 0 for less challenging and 1 for more challenging. Statistically reliable effects (p<0.05) are indicated with bolded p values. Marginally reliable effect (p<0.07) are indicated with a single asterix (*).

### Statistical procedures

Our data consisted of a hierarchically structured combination of fixed (e.g., song playback treatment) and random (e.g., chamber) effects, which may differ from one another in their correlation structure. Therefore we analyzed these data in a mixed, multilevel modeling framework using the software R 2.7.2 [74], which readily accommodates hierarchically structured combinations of fixed and random effects. We included the level of song challenge (i.e., the playback treatment) and the number of songs a male produced on the final morning before sacrifice as predictors in all models. We ran one model for each compound predicted to be of importance in each of the brain regions of interest. We used a general linear mixed model (GLMM; nlme package; [75]), which uses t-tests to test the null hypothesis that a coefficient equaled 0. We estimated parameters with restricted maximum likelihood (REML) and we modeled chamber as a random intercept and random coefficient on playback treatment in all cases. The song playback treatment in all models was coded 0 for less challenging and 1 for more challenging.

## Results

**Figure 4:**
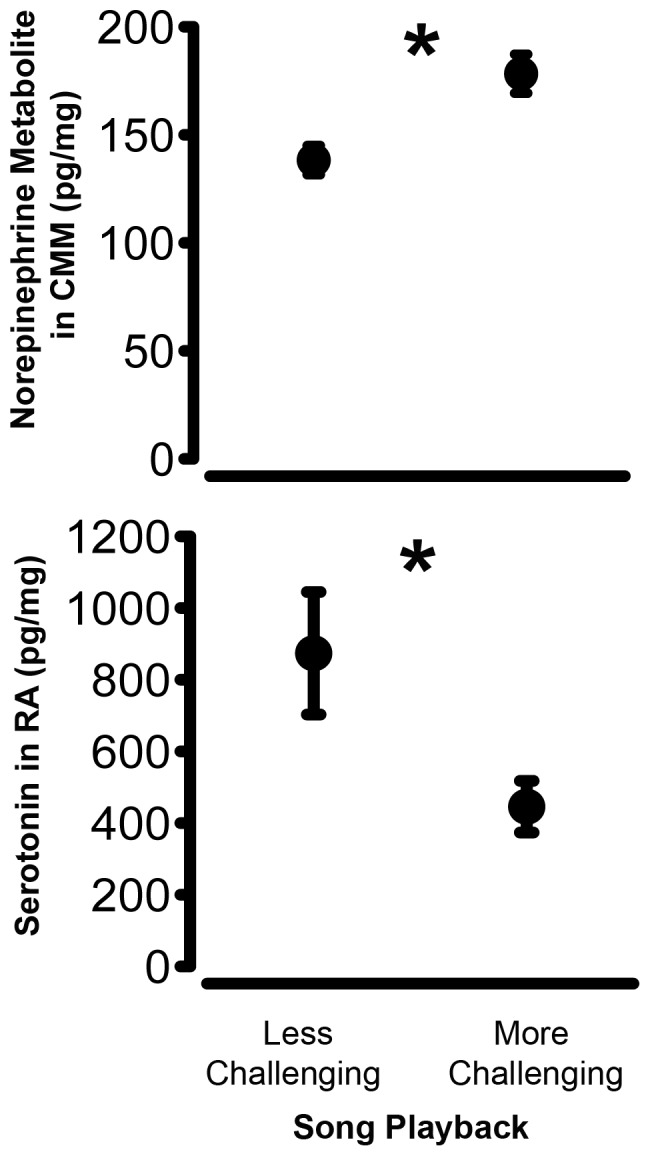
Treatment effects on forebrain monoamines. The effects of the level of song challenge on the amount (mean pg/mg of protein ± SEM) of (a) norepinephrine metabolite in the caudomedial mesopallium (CMM) and (b) serotonin in the robust nucleus of the arcopallium (RA).

Males that had been exposed to more challenging songs for a week had higher levels of norepinephrine metabolite in an auditory processing region, the CMM, relative to males exposed to less challenging songs [GLMM, effect of level of song challenge, t = 4.280, p = 0.008; Fig. 4a; Table 1]. Additionally, males that had been exposed to the more challenging song treatment had lower levels of serotonin in RA [GLMM, effect of level of song challenge, t = −2.657, p = 0.038; Fig. 4b; Table 1]. For all the other compounds in all the other brain regions examined, we were unable to find reliable differences based on the level of song challenge males experienced for a week (all p>0.05, Table 1).

We did not find any statistically significant relationships between a male's own singing effort the morning before sacrifice and the level of any compound of interest in any brain region examined (all p>0.05). However, because the playback treatment was positively associated with singing behavior, it cannot be ruled out that self-stimulation from a male's own singing may have contributed to the observed differences and future studies should evaluate this potential contribution to the observed treatment effects. Nonetheless, the present results indicate that the level of song challenge caused changes in monoamine and metabolite levels that cannot be explained by recent singing behavior.

## Discussion

Male Lincoln's sparrows exposed to more challenging songs shift their competitive behavior to sing more over the period of a week [16]. We argue that the gradual change in behavior and the persistence of this behavioral difference on the day after playback ceased reflects a shift in the males' competitive state as a function of longer-term social conditions. Here, we show that males exposed to more challenging songs also had higher levels of norepinephrine metabolite, suggesting higher levels of norepinephrine breakdown, in a perceptual brain region specifically implicated in song discrimination and recognition, the CMM ([76], [77]; Fig. 4a). Additionally, these males had lower levels of serotonin in the principal motor nucleus within the song control pathway, RA (Fig. 4b). There were no reliable relationships between levels of monoamines or their metabolites and singing output immediately prior to sacrifice. Further, the relationships between the level of song challenge and the levels of monoamines and their metabolites was independent of the level of recent singing effort, indicating that the detected differences in monoamine levels could not be explained by fluctuations in this recent behavior. The association between some monoamines and the social treatment, combined with our previous report that this social treatment induces changes in competitive behavior [16] is consistent with the established role of the monoamines as modulators of sensory and motor processes underlying adaptive shifts in behavioral state. Collectively, these findings demonstrate that persistent variation in male-male competition reflected by the level of song challenge elicits concerted changes in at least two important monoamine neuromodulator systems within both perceptual and motor control brain regions.

The monoamines largely mediate neural plasticity underlying shifts in behavioral state by influencing the sensitivity or excitability (i.e., cellular properties) of target neurons [1], [7], [37]. Differences in behavioral state can be regulated by differences in monoaminergic activity over extended periods. For example, shifts in attention and motivation, differences in mood, mood disorders and behavioral pathologies such as schizophrenia are all associated with long-term differences in forebrain monoamine levels [2], [9], [78]. However, transient differences in monoamine levels may also alter synaptic properties and neural connectivity within the forebrain [1], [2], [78], [79] and the present experimental design would not have captured such transient actions of the monoamines. Thus, it is probable that there were additional differences in monoamine levels in the days prior to our measurements, and that some of these undetected monoaminergic differences contributed to neural plasticity and the ultimate behavioral shift that was observed, making the reported findings a conservative summary of treatment effects. Similarly, the experimental design could not discriminate between persistent differences and effects within the final day of the manipulation and thus we cannot know how long the differences we did detect may have persisted. Despite these caveats, the present study did find differences in the levels of monoamines and their metabolites in perceptual and song motor control brain regions, indicating that some monoaminergic changes occur in response to longer-term social conditions. Future studies manipulating monoamine levels across social contexts and timelines are needed to demonstrate if and how the differences detected in the present study are causally tied to changes in behavioral state, and to determine the timelines of such brain changes.

### Effects of the level of song challenge on monoamines in regions of the auditory telencephalon

Neural activity in the NCM and the CMM occurs in response to hearing conspecific songs [44], [80], [81], varies as a function of qualitative differences among songs [76], [77], [82]–[84] and is influenced by recent experience [72], [85] and context [86]. The NCM in particular shows strong staining for dopamine beta hydroxylase, leading to the inference of strong noradrenergic innervation, likely from the locus coeruleus [87]. Across vertebrate taxa, norepinephrine plays a central role in focusing attention on relevant stimuli [4], [7] and in improving perceptual acuity in sensory brain regions [1], [7] including auditory processing regions [8], [39]. In birds, ablating noradrenergic inputs to the forebrain abolishes biased behavioral [38], [88] and neural [35] responses in the NCM and the CMM, to preferred signals. Further, exposure to persistent song playback affects norepinephrine secretion and metabolism in the auditory forebrain of female birds [36]. Collectively, this evidence supports the role of norepinephrine in modifying the sensitivity of neurons within these auditory brain regions as a function of social conditions, perhaps by increasing neural responsiveness to relevant cues [35].

Based on previous studies, the present finding of higher norepinephrine metabolite levels, and thus presumably norepinephrine metabolism, in the CMM of male birds exposed to more challenging songs (Fig. 4a) could reflect increased sensitivity and attention to song challenge [35], [38]. The absence of a concomitant increase in norepinephrine secretion in the NCM may be surprising because the NCM is implicated in the processing and memorization of song [89] and noradrenerigic activity is specifically implicated in this neuronal adaptation [90]. However, neurons in the NCM respond to novelty and neuronal activity in this brain region decreases with habituation [41], [81], [91]. Thus, it is possible that any differences between treatment groups in noradrenergic activity in the NCM occurred very quickly [90] as auditory memories were encoded and treatment differences in NCM were not detected due to the experimental timeline. It is equally possible that males in the two treatments groups, having been exposed to song playback for the same duration of time, encoded those auditory memories with equal fidelity despite the apparent difference in the saliency of the stimuli. In contrast to the NCM, CMM neurons may respond to familiar songs ([77], though see [81]). Given that the subjects had been exposed to the same set of recordings for 7 days, presumably making them familiar, it seems reasonable to anticipate greater changes in the CMM in response to such persistent challenge. Determining if and how norepinephrine secretion and metabolism in the CMM could in turn affect males' behavioral output will require manipulations of norepinephrine levels in the auditory forebrain. Because the caudomesopallium contains neurons that project to a central nucleus of the song control pathway (HVC) or the nearby nidopallium [92], [93], it is reasonable to hypothesize that norepinephrine's effects in the CMM could ultimately influence song output.

### Effects of the level of song challenge on monoamines in nuclei of the song control system

Persistent playback of more challenging song reduced levels of serotonin in the principal nucleus of the song motor control pathway, RA, relative to playback of less challenging song (Fig. 4b). Nucleus RA, in concert with area X, is implicated in context-specific singing behavior [55], [56] that occurs on a temporal scale ranging from seasonal shifts in song output [94]–[96] to moment-to-moment changes in song quality associated with the presence of a female [55], [56]. While area X is thought to regulate shifts in the quality and stereotypy of song, the RA translates pre-motor signals from HVC and the anterior forebrain pathway into coordinated movements of the respiratory and syringeal muscles [55], [56], [97]. Both RA and area X receive catacholaminergic inputs from the dopaminergic center, the ventral tegmental area (VTA; [11], [37], [49]–[54]); and serotonergic innervation of the entire avian forebrain from the raphe nuclei is extensive [57].

There is strong evidence that neural activity in the VTA regulates context-specific activity in area X, and therefore RA [11], through dopaminergic inputs and that dopamine levels in these regions ultimately control song output [47], [48], [55], [56], [98]–[104]. However, the effect of the social treatment on dopamine levels in RA fell just short of statistical significance in the present study (p* = *0.055; Table 1). Nor was there good evidence that singing immediately prior to sacrifice was correlated with levels of dopamine or its primary metabolite in area X (p = 0.113; Table 1). The absence of detectable variation in dopaminergic activity in area X, the brain region most frequently implicated in context-specific singing [11], is surprising but could be explained by the fact that previous studies of singing modulation focused on short-term mate attraction efforts in colonial birds (e.g., [56]). In contrast, the present study examined dopaminergic responses to persistent signals of male-male competition (not mate attraction) in a territorial species; subjects were exposed to equal durations of song playback that differed in the relative level of social challenge it reflected. Subjects did not differ in the quality of songs that they produced [16], a feature of singing behavior associated with shifts in mate attraction efforts and neural activity in area X [55], [56]. However, males did differ in the amount of song they produced, a measure associated with shifts in competitiveness, territoriality [29], [30], [32], [105], and neural activity in RA [55], [106], perhaps explaining the marginal treatment effect on dopamine levels in RA (Table 1). Though the present results are not robust, they do encourage future study of the effect of dopamine manipulations in RA on the rate of singing in territorial birds.

Given the role of RA in regulating song output, we expected that monoaminergic differences in this brain region would be correlated with recent motor output of song, independently of treatment. In particular, we expected levels of dopamine and serotonin metabolite to correlate positively with recent singing [47], [48], [62]–[64], [100], [101], [104], [107]. However, surprisingly, the most robust finding in this brain region was that the level of serotonin was explained by the level of song challenge (p = 0.038; Fig. 4b) and was not reliably associated with a male's own singing behavior in the hours before sacrifice. This result supports the conclusion that serotonin was differentially regulated within RA as a function of social experience. Given that there was no treatment effect on serotonin metabolite, elevated serotonin might be interpreted to reflect increased presynaptic levels or higher extracellular levels of this monoamine. Such a pattern could result from increased synthesis and sequestration presynaptically, or decreased activity of catabolic and re-uptake enzymes (e.g., monoamine oxidases and transporters) leaving more serotonin in the synapse. Though the absence of a concomitant treatment effect on serotonin metabolite is difficult to reconcile, increased serotonergic activity (i.e., decreased metabolite levels and in some cases decreased serotonin levels [108]) is associated with increased aggression across vertebrate species [15], [65], and singing behavior in the context of male-male competition is an aggressive behavior. In the present study, though we did not find an effect on metabolite, we did find decreased serotonin levels in males that were exposed to more challenging songs, who sang more, and were thus inferred to be in a more competitive behavioral state. Thus, the present findings are not completely inconsistent with the broader body of research on serotonergic regulation of aggression.

In addition to regulating aggressive behavior, serotonin is also reported to regulate vocalizations across taxa [62]–[64]. For example, pharmacological inhibition of serotonin reuptake (and thus presumably an elevated level of serotonin) is reported to suppress vocalization rate in several species [62]–[64]. This prior work is consistent with the present finding that males exposed to less challenging song, who sang less, had higher serotonin levels (though whether serotonin was elevated within the synapse or presynaptically cannot be determined in our study). Future studies manipulating serotonin levels and examining behavioral response to social challenge over time will clarify how serotonin might contribute to changes in competitive singing.

## Conclusions

Collectively, the present data demonstrate that persistent differences in the level of song challenge known to elicit a change in competitive behavioral state in territorial male songbirds, also affect monoaminergic measures in perceptual and song motor control brain regions. These findings implicate monoamine-induced neural plasticity in achieving adaptive changes in behavioral state in response to longer-term shifts in social conditions. Further, they support the hypothesis that social cues affect multiple brain regions in different but perhaps coordinated ways to ultimately achieve adaptive shifts in behavior. Future manipulative experiments building upon these findings will elucidate the causal relationships between monoaminergic activity and socially-induced changes in behavioral state. As our understanding of the monoamine systems increases, there is ever growing need to examine concerted changes across neuromodulatory systems, interconnected brain regions, and timelines, in relation to environmental conditions and associated behavioral outcomes [109], [110]. Conducting this work in diverse wild species with different natural histories also contributes to our understanding of how selection processes have shaped the concerted brain mechanisms underlying the adaptive modulation of behavior in response to changing conditions.
